# The Comet Assay: Automated Imaging Methods for Improved Analysis and Reproducibility

**DOI:** 10.1038/srep32162

**Published:** 2016-09-01

**Authors:** Signe Braafladt, Vytas Reipa, Donald H. Atha

**Affiliations:** 1 Biosystems and Biomaterials Division, National Institute of Standards and Technology, Gaithersburg, MD 20899, USA.

## Abstract

Sources of variability in the comet assay include variations in the protocol used to process the cells, the microscope imaging system and the software used in the computerized analysis of the images. Here we focus on the effect of variations in the microscope imaging system and software analysis using fixed preparations of cells and a single cell processing protocol. To determine the effect of the microscope imaging and analysis on the measured percentage of damaged DNA (% DNA in tail), we used preparations of mammalian cells treated with etoposide or electrochemically induced DNA damage conditions and varied the settings of the automated microscope, camera, and commercial image analysis software. Manual image analysis revealed measurement variations in percent DNA in tail as high as 40% due to microscope focus, camera exposure time and the software image intensity threshold level. Automated image analysis reduced these variations as much as three-fold, but only within a narrow range of focus and exposure settings. The magnitude of variation, observed using both analysis methods, was highly dependent on the overall extent of DNA damage in the particular sample. Mitigating these sources of variability with optimal instrument settings facilitates an accurate evaluation of cell biological variability.

Many studies in the area of genotoxicology use cytotoxicity measurements as a proxy to reflect DNA damage. However, cytotoxic effects often occur at high toxicant dose and the subtler effects that may arise prior to reaching the cytotoxic threshold are mechanistically interesting. One of the first effects observed in cells subjected to oxidative stress is damage to DNA. The comet assay, also referred to as single-cell gel electrophoresis (SCGE), has been extensively used in assessing genotoxic effects in cells exposed to various toxicants, including nanomaterials^1^,^2^,^3^,^4^. The comet assay is a sensitive and efficient technique for analyzing DNA damage in cells, caused by strand breaks, DNA lesions, alkali labile sites and DNA cross-linking with protein^5^,^6^. It is based on the migration of cleaved DNA out of the nuclei in the electric field, with the intact DNA remaining within the nucleoid. Analysis of the DNA “comet” tail and nucleoid shape as well as the migration pattern allows for relative assessment of DNA damage. Our previous studies have implemented the comet assay for monitoring the genotoxic effects of cadmium selenide nanoparticles on normal human bronchial epithelial (NHBE) cells^7^.

The comet assay offers sensitive detection of both single and double stranded breaks. However, there are possible sources of variability in the assay that affect its reproducibility. This includes the multiple steps involved in processing the cells to be analyzed, the electrophoresis and the staining procedure^8^,^9^. The inherent heterogeneity of the cells used in the comet analysis requires that a statistically relevant number of cells must be examined to obtain a representative average. This becomes practical only using an automated system for data collection and analysis^10^. Another source of variability is related to the imaging and analysis procedures required to quantify the results. It is important to establish optimal parameters for image data collection before the comet assay can be used in routine studies. In this study we focus on the sources of variability in the imaging and analysis of comet assay samples using public domain and commercially available software. We compare the use of the automated features of the commercial software with image and scoring measurements using the public domain software. In addition, we demonstrate the utility of automated image capture to increase the number of comet images and improve statistics, following etoposide and electrochemical oxidative treatments of the cells.

## Methods

### Materials

A reference kit of human leucocyte cells with varying levels of etoposide^11^ treatment (Cat. No. 4256-010-CC) was obtained from Trevigen Inc. (Gaithersburg, MD, USA) and stored at-150 °C. Stock cultures of Chinese Hamster Ovary cells, CHO K1 (ATCC, Manassas, VA, USA) were grown at 37 °C, 5% CO_2_ and 90% relative humidity in Dulbeco’s modified eagle medium (Gibco, Carlsbad, CA), 10% (vol. frac.) fetal bovine serum, FBS (Gibco), 1% (v/v), penicillin-streptomycin (100 units/mL, and 100 μg/mL).

### Electrochemical Cell Oxidation

A uniform oxidative stress was simulated by growing the CHO cells on electrode surfaces polarized at a fixed potential. Indium tin oxide films on glass (Delta Technologies, Loveland, CO) were used as transparent electrodes, placed in 80 mm diameter glass Petri dishes with Pt wire as a counter electrode and a Ag/AgCl reference electrode (Microelectrodes, Inc.). The transparent electrodes were cleaned by sonicating 15 min in hot 50% (vol. frac.) ethanol/water mixture, followed by air drying prior to mounting them in Petri dishes. Contacts to the conducting film surface were provided by wire contact using an InGa eutectic and were insulated with waterproof silicone. Cells were grown on these indium tin oxide electrodes at open circuit conditions until they reached confluence, and the electrode potential E = +1V (vs Ag/AgCl) was applied using an EG&G Princeton Applied Research 363 potentiostat. Prior to culture, the electrodes were coated with fibronectin (25 μg/mL in Dulbeco’s phosphate buffered saline (Gibco), DPBS for 1h at room temperature). The cell oxidative exposure level was varied by varying the duration of the electrode polarization between 6 h to 12 h. At least three replicates were produced at each treatment. The cells were harvested after treatment by gentle rinsing of the electrodes with DPBS and placing in a clean Petri dish. Trypsin EDTA (2 mL of 2.5 mg/mL) was added for 3 min to 5 min at 37 °C to detach the cells. After microscopic verification of cell detachment, the trypsin treatment was stopped with the addition of 10 mL medium and the suspended cells collected and concentrated by centrifugation at 250 × g_n_ for 5 min at 4 °C.

### Comet Assay

DNA fragmentation was measured by the alkaline comet assay, which is known to detect both single and double strand breaks in a wide variety of cell types^6^. Low melting point agarose, 300 μL (Trevigen Inc.Cat. No. 4250-050-02) was heated to 37 °C and combined with 30 μL of a 1 × 10^5^ to 2 × 10^5^ cells per mL cell suspension (ratio 1:10 volume fraction), determined using a hemocytometer (Fisher Scientific, Inc.). Each well of a 20-well CometSlide (Trevigen Inc. Cat. No. 4252-200-01) was filled with 30 μL of the cell/agarose suspension. The slides were placed in a 4 °C refrigerator in the dark for 15 min to solidify. Slides were then immersed in 50 mL of pre-chilled lysis solution containing Trizma base, Triton X-100, DMSO (Trevigen Inc. Cat. No. 4250-010-01) and left at 4 °C for 30 min to facilitate cell membrane and histone removal. After draining excess liquid, the slides were transferred to 50 mL of freshly prepared (same day) alkaline DNA unwinding solution, (200 mmol/L NaOH, 1 mmol/L EDTA, pH > 13) and incubated at room temperature in the dark for 20 min. After the unwinding step, electrophoresis was performed in the CometAssay ES tank (Trevigen Inc.) at 21 V for 30 min. Slides were then rinsed with distilled water and fixed 5 min in 70% ethanol. Slides were dried and stained 5 min at 4 °C with SYBR Green I (Trevigen, Inc., Cat No 4250-050-05) diluted 1:10 000 in 10 mmol/L Tris pH 7.5, 1 mmol/L EDTA, drained to remove excess staining solution and thoroughly dried at room temperature in the dark.

### Microscopic Image Analysis

Slides were visualized by epifluorescence microscopy using an Olympus BH-2 System microscope equipped with Olympus SPlan 10 and DPlanApo 20 UV objectives, an optical filter set for SYBR Green I (excitation/emission wavelength, 460 nm and 560 nm respectively, Chroma, 49002 ET GFP), a LUDL MAC 6000 automated stage (Hawthorn, NY, USA) and a Photometrics (Tucson, Az, USA) Snapcool HQ2 monochrome CCD camera using NIKON Basic Elements software ver. 4.20.01. Images were captured (32 frames per each 1 cm diameter slide well) and the stage controlled by the NIKON Elements software. Integrated intensities and % DNA in tail were determined using Image J ver. 1.47v (public domain) and TriTek (Sumerduck, VA, USA) CometScore Pro ver. 1.01.44 commercial software utilizing the following equations:


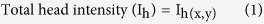



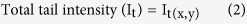



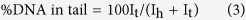


where I_h(x,y)_ and I_t(x,y)_ are the individual pixel intensities within the head and tail regions of the comet image. Image J is semi-automated image analysis software written under NIH contract and is in the public domain^12^. It uses many standard image analysis routines that can be used to perform calculations for a wide range of applications, including comet image analysis. CometScore Pro is commercially available software, which has been specifically developed to automate comet image analysis. The software includes both automatic and manual scoring of comets and produces a listing of each scored comet and its corresponding image. Overlapping comets and artifacts are eliminated from the list prior to calculation of the mean percentage DNA in tail.

To examine sources of variability in the comet imaging and analysis we used a microscope slide containing comets from cells with various levels of DNA damage. The Trevigen reference kit (Sample CC3) contains a population of comets ranging from control (no strand breakage) to large tail comets (high strand breakage). A slide containing these comets was used in this study to analyze the effect of camera exposure and focus without the need to change the microscope slide position. To examine the distribution of etoposide produced comets, we used the automated microscope system to examine individual slides containing each of the four cell samples (CC0, CC1, CC2 and CC3) provided in the Trevigen reference kit. For the electrochemically produced comets we used slides from comet analysis of the CHO cells grown on indium tin oxide electrodes.

### Disclaimer

 Certain commercial equipment, instruments and materials are identified in this paper to specify an experimental procedure as completely as possible. In no case does the identification of particular equipment or materials imply a recommendation or endorsement by the National Institute of Standards and Technology nor does it imply that the materials, instruments, or equipment are necessarily the best available for the purpose.

## Results

**Figure 1:**
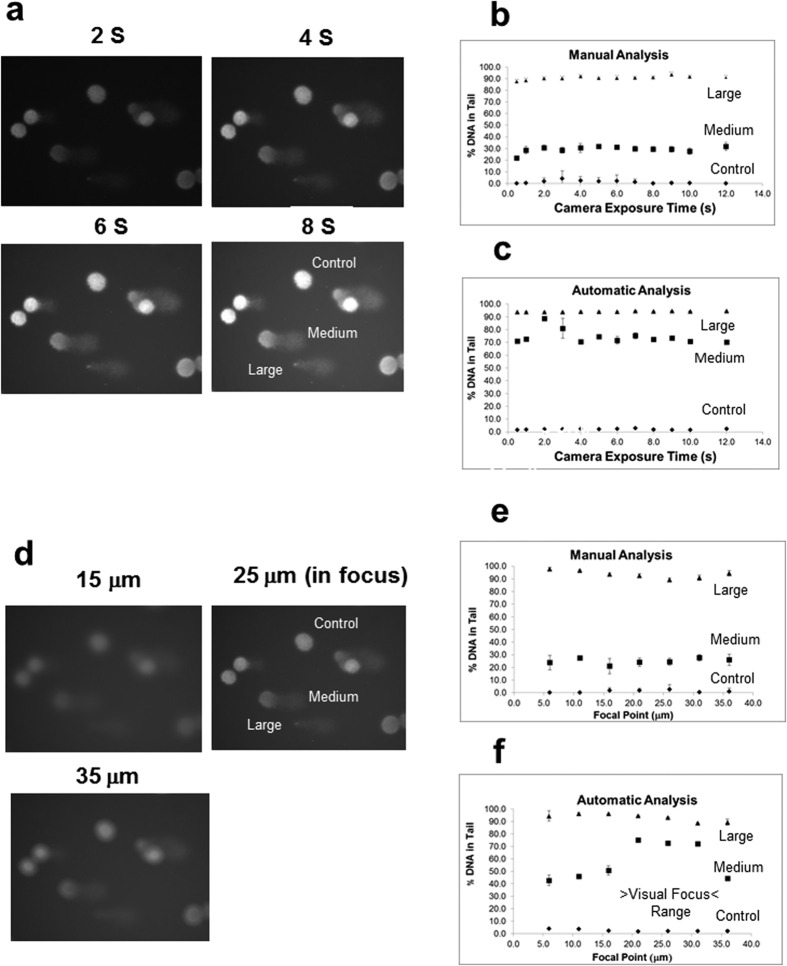
Effect of Camera Exposure Time and Focus on Comet Image Analysis. **(a)** Example of comet images taken at 2 s to 8 s exposure showing, Control (no strand breakage) Medium (medium strand breakage) and Large (high strand breakage) tail comets. **(b)** Manual scoring analysis of single control, medium and large tail comets; **(c)** Automatic scoring analysis of single control, medium and large tail comets. The error bars indicate the standard deviation in the %DNA in tail for each camera exposure time measurement (n = 5). **(d)** Example of comet images taken at three microscope z axis focal points (15 μm, 25 μm, and 35 μm). 20 μm to 30 μm was considered to be within the visual focus range. **(e)** Manual scoring analysis of single control, medium and large tail comets. **(f)** Automatic scoring analysis of single control, medium and large tail comets. The error bars indicate the standard deviation in the % DNA in tail for each camera exposure and focus measurement (n = 5).

Figure 1a–c show the effect of camera exposure time on the calculated percent DNA in tail of the single control, middle size and large tail comets taken from the same slide. We examined one comet image at a time in order to examine the variability due to camera exposure and focus in well-defined images.

These images were analyzed five times at each camera exposure using the manual and automatic scoring methods of the commercial software. For control and large-tail comets, both the manual and the automatic methods were consistent and reproducible with negligible variation within a limited range of camera exposure time. This indicates that the commercial imaging software correctly compensated for the expected increase in background intensity with increased camera exposure time. This also indicated that significant photobleaching of the Sybr Green dye did not occur in the range of exposure times used. However, the medium-size comet shows a large discrepancy between the manual and automatic analysis methods due to the difficulty in accurately determining the intensity centroid of the comet head using the manual method. This led to a large difference in the calculation for the percentage DNA in tail for these comets. As long as the overall intensity is high enough (4 s to 12 s exposure time) the automatic method compensates for the intensity difference within the nuclear region of the medium sized comets and yields a more accurate determination of the tail and head regions.

Figure 1d–f show the effect of microscope camera focus on the measured percent DNA in tail of single control, medium size and large tail comets. Again, the control and large-tail comet measurements yielded consistent and reproducible results for the manual and automatic methods. However, the data for the medium-size comets are only stable and consistent with the previous automatic measurements (Fig. 1c) in the focal point Z-axis range of 20 μm to 30 μm. This is a result of the failure of the automatic mode of the commercial imaging software to accurately determine the intensity centroid of the medium-size comet unless the microscope camera is sufficiently focused.

**Figure 2:**
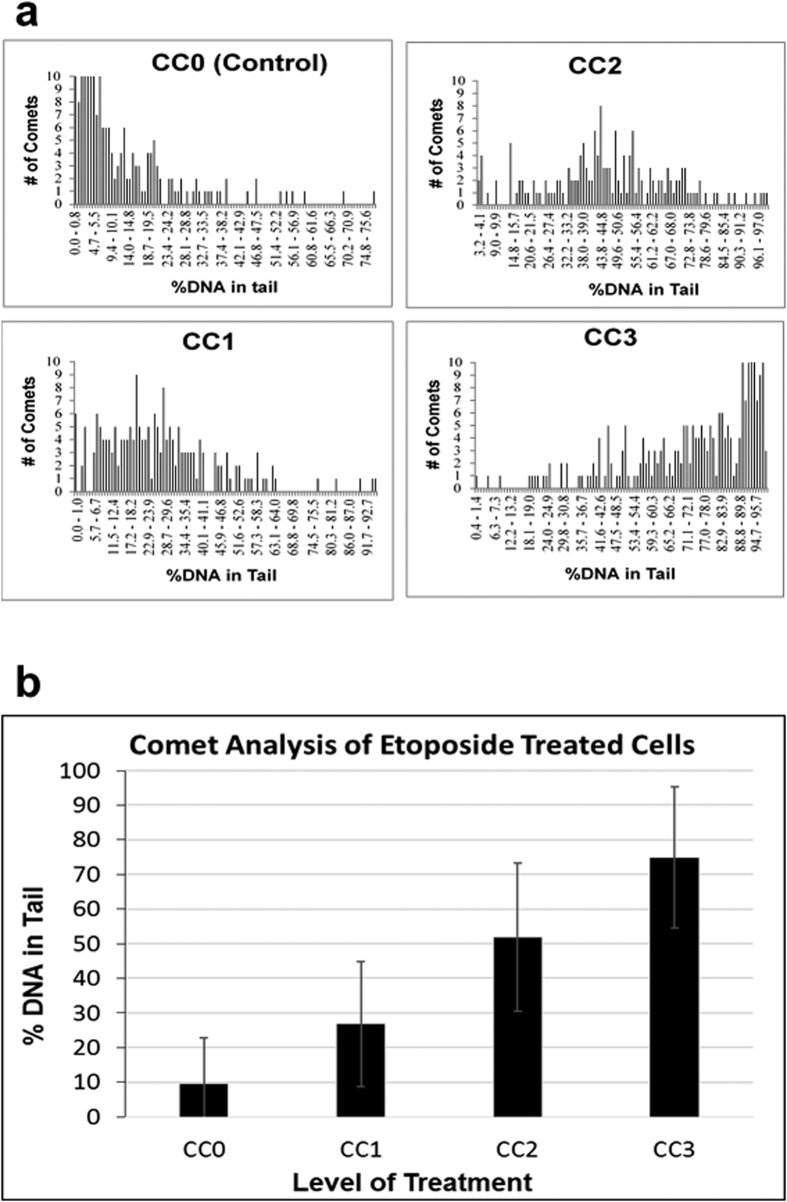
Analysis of Etoposide Treated Cells. **(a)** Graphical representation of the distribution of the comets after etoposide treatment. The number of comets within each bin (% DNA in Tail) is plotted for each of four chemical treatment levels (CC0, CC1, CC2, CC3). **(b)** Comet analysis at the four etoposide treatment levels showing average % DNA in tail for one set of measurements. The error bars indicate the polydispersity of the individual comets for each treatment level.

Figure 2a shows the result of automated image analysis of four reference preparations of the etoposide treated human leucocytes in a 20 well slide (CC0, CC1, CC2 and CC3). The number of non-overlapping comets used in the analysis ranged from 401 for the control (CC0) to 313 for the treated (CC3) cells. To test reproducibility, the slide configuration is set up such that each preparation is pipetted into a separate row vertically and with equivalent samples pipetted horizontally across the five wells in each column. For each treatment level the polydispersity of the comets is shown graphically as a distribution with respect to % DNA in tail. The bar graph in Fig. 2b shows the average % DNA in tail at each treatment level for one set of measurements. The large error bars demonstrate the high polydispersity of the individual comets. The slide well-to-well reproducibility for the five wells was also measured for each of the four reference samples of etoposide treated cells (Supplementary Fig. 4). The variation in the means of the individual wells were about one third of standard deviations shown in Fig. 2b. This indicated that the well-to-well measurements (n = 5) were within the expected variation of the means (σ/n^1/2^) and did not indicate additional sources of variation such as could occur with pipetting (i.e., a non-uniform agarose cell suspension).

**Figure 3:**
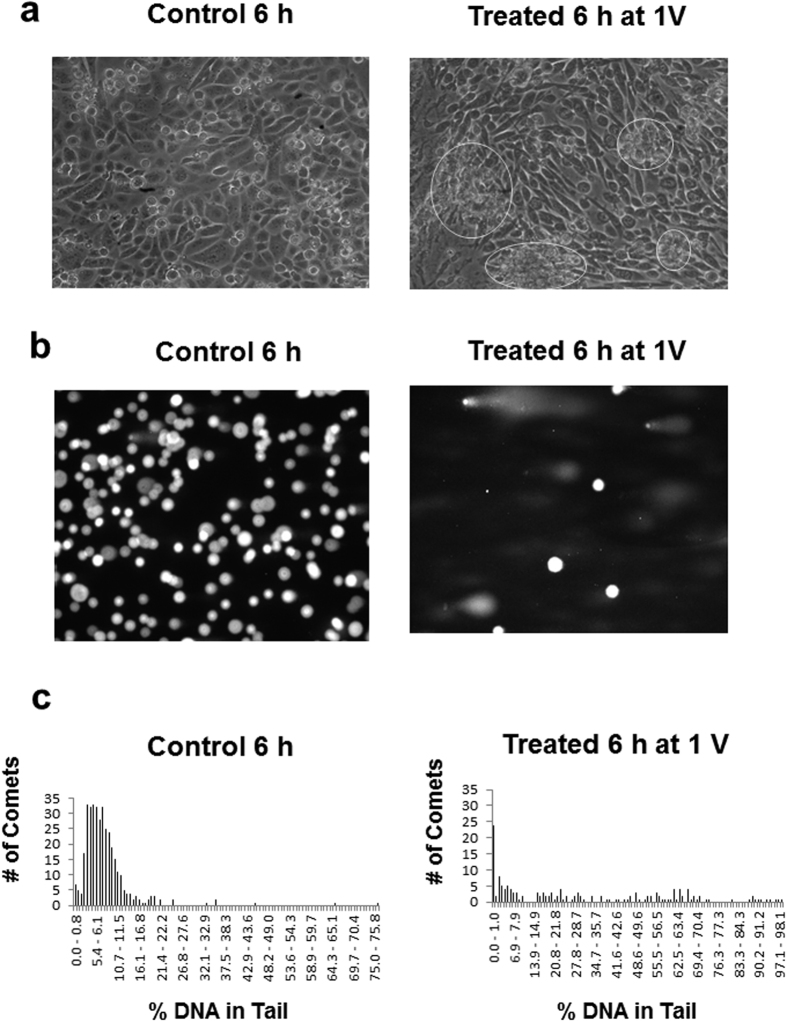
Analysis of Electrochemically Treated Cells. **(a)** Images of CHO cells grown on indium tin oxide electrode. Images are shown comparing control (untreated) and electrochemically treated (6 h at 1V) CHO cells during culture. Clusters of affected cells are indicated by circular regions. **(b)** Images are shown comparing comets from control (untreated) and electrochemically treated CHO cells. **(c)** Distribution of comets after Electrochemical Treatment (Treated 1). Graphical representation showing number of comets as a function of % DNA in tail for control (untreated) and electrochemically treated CHO cells.

Figure 3a shows the microscopic images of cultured CHO cells grown on indium tin oxide electrodes, comparing the control (open circuit during growth) with cells, exposed to E = 1 V for 6 h. These treatment conditions were chosen, based on previous experiments to yield cells with significant DNA damage. The resulting cells are used here to demonstrate the ability of the commercial software to analyze a very heterogeneous population of comets where simple averaging might yield an incomplete picture. The treated cell image shows that a few patches of tightly clustered cells, (as indicated by circled regions) are beginning to develop and may be an effect due to the oxidative stress. The resulting comet images are shown for comparison in Fig. 3b. The calculated distributions of the individual comets are presented in Fig. 3c. The treated sample image shows a much lower population of normal cells and a high percentage of cells with varying degrees of damaged DNA. The mean and standard deviation calculated for the control and treated populations are presented in Table 1. The polydispersity of the treated-cell comets is so high that the graphical representation of the distribution of comet sizes, expressed as % DNA, is a more complete and useful representation, as evident from Fig. 3. None-the-less, when two separate analyses were performed on the same treated sample (Treated 1 and Treated 2), the means were not found by the t-test to be significantly different (p < 0.05).

**Figure 4:**
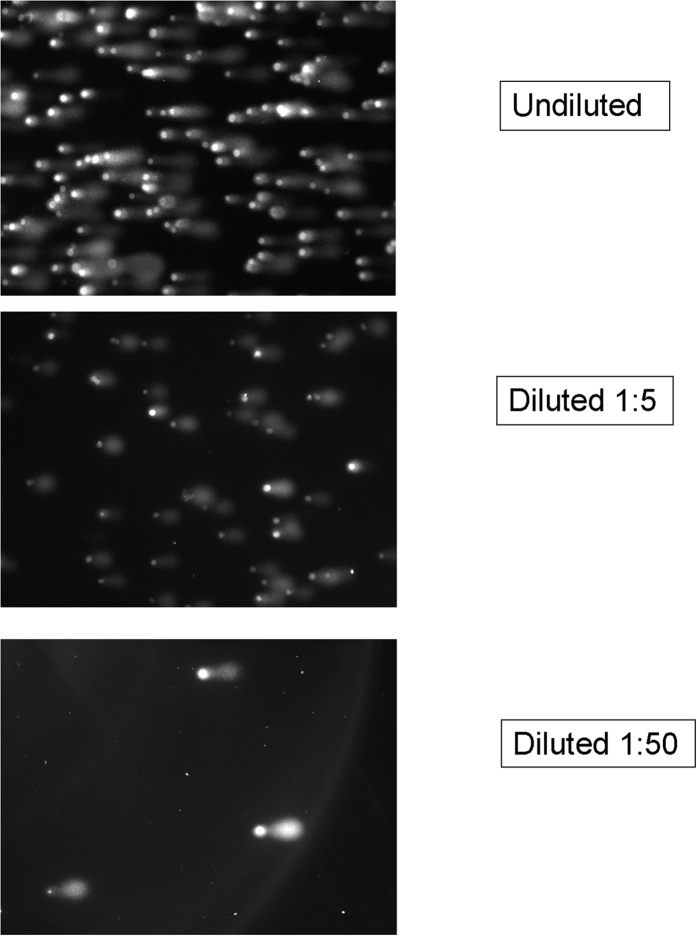
Effect of cell concentration on comet analysis. Typical comet images using samples with decreasing cell concentrations (from ≈10^5^ cells per milliliter to ≈10^3^ cells per milliliter). Cells were obtained after electrochemical treatment, 1 V for 12 h.

The effect of cell concentration on the comet image analysis was examined in Fig. 4 For these measurements the comet image analysis was performed on three cell concentrations ranging from 10^5^ cells per milliliter to 10^3^ cells per milliliter (final concentration in the agarose mixture). A preparation of electrochemically treated cells (12 h at 1 V vs Ag/AgCl) was used for this purpose. Comet analysis was performed on undiluted cells and at dilutions of 1:5 and 1:50). The cell density used in the undiluted sample was about 10 fold higher than usual for the agarose suspension. In this case about 50% of the comets were overlapping and were not counted in the analysis. The comparison to lower cell densities (at 1:5 dilution about 20% of cells overlap) demonstrated that the average percent DNA in tail and standard deviation remained essentially the same up to a 50 fold dilution, where overlapping is negligible. Table 2 shows that throughout this range, the commercial comet analysis software yielded consistent results.

## Discussion

The measurements in this study have focused on the effect of imaging parameters on comet assay analysis. Specifically, the effect of microscope camera and imaging software settings were examined using a select set of images produced using a fixed comet assay procedure. Establishing the effect of imaging parameters will be critical in future examination of the individual steps involved in the comet assay procedure (sample cell preparation, agarose formulation, cell lysis, DNA unwinding, electrophoresis and staining)^8^,^9^.

In this study, we found that our camera system exhibited linearity of the control comet intensity within the 1 s to 12 s range of exposures used in our experiments (Supplementary Fig. 2). This linearity is essential to ensure that the ratio of integrated intensities of the nuclear and tail regions of the comet properly reflect the proportion of DNA in the tail. A fluorescence intensity reference standard will be useful in future lab-to-lab comparisons to ensure that the microscope light source and camera sensitivity settings are in a range to obtain this linearity^13^. The absolute determination of percentage DNA in the tail is also dependent on sequence specificity of fluorescent dye binding, differences in dye binding to whole and damaged DNA and to differences in binding to single and double-stranded DNA as well as fluorescent quenching effects. For example, Sybr Green I has been observed to bind single-stranded DNA with about an 11-fold lower affinity than double-stranded DNA^14^. In addition, sequence specificity and salt effects were also observed^14^. Quantifying each of these effects is outside the scope of this study. However, with a well-characterized image system in place, the effect of staining conditions such as type of dye, concentration, temperature and ionic strength can be thoroughly examined.

The integrated intensity of a single control cell comet was consistent between the public domain and the commercial imaging software analysis (Supplementary Fig. 1 and Supplementary Tables 1 and 2). However, when calculating the % DNA in tail of a medium size tail comet, we found that the measurement becomes very sensitive to the intensity threshold setting in the public domain software. For both software types, the threshold was set right at the point where the image background is eliminated. This manual threshold setting resulted in constant values of % DNA in tail for control and large tail comets. We also determined that the photobleaching of the sample from the microscope light source up to 12 s exposure did not significantly affect the % DNA in tail measurements. This is consistent with the uniform % DNA in tail measurements at high camera exposure times (Fig. 1b,c). It should be noted that the dye photobleaching rate needs to be carefully calibrated for each microscope system due to lightsource intensity variation (Supplementary Fig. 3).

When using the commercial comet analysis software, the integrated intensities of a single camera exposure time and focus setting were consistent between the manual and automatic modes for the control and large tail comets. However, the medium tail comets exhibited inconsistent measurements dependent on the exposure time. Variations as high as 40% were observed between manual and automatic modes (Fig. 1b,c). This was due to the difficulty to precisely determine the mean integrated intensity center line for the comet head using the commercial software in the manual mode. In the manual mode only an estimate of the head center can be made. This effect was verified by comparing the % DNA obtained in the manual mode of the commercial imaging software with slight changes in the position of the curser, which is used to determine the center of the comet head. However, in the automatic mode, when used within specified ranges of instrument settings (camera exposure time 4 s to 12 s) the variation was reduced. Camera exposure range would depend on the intensity of the comet images (i.e., dye staining, sensitivity of the particular camera system, light source intensity, etc.). Within the focus setting of 20 μm to 30 μm, measurements were consistent between the automatic focus and exposure time measurements. Under these conditions the automated image analysis software can provide reproducible measurements of % DNA fragmentation over a wide range of comets (Fig. 1c).

We also found that the commercial comet analysis software yielded reproducible data when used to analyze images over a wide range of cell densities. Even over a 50-fold cell concentration range (≈10^3^ cells per milliliter to 10^5^ cells per milliliter), the resulting % DNA fragmentation was nearly identical (Table 2). The cell density used in the undiluted sample was about 10 fold higher than usual for the agarose suspension. In this case about 50% of the comets were overlapping and were not counted in the analysis. The comparison to lower cell densities (at 1:5 dilution about 20% of cells overlap) demonstrated that the average percent DNA in tail and standard deviation remained essentially the same up to a 50 fold dilution, where overlapping is negligible. Since the population of comets in this highly treated sample was fairly homogeneous, the elimination of overlapping comets yielded very little change in the average percent DNA in tail. However, at high cell density, in a highly heterogeneous population of comets, the elimination of overlapping comets would be expected to lower the number of large tail comets and thus reduce the average percent DNA in tail. This effect is minimal at our typical density of 10^4^ cells/milliliter (final concentration in agar) where less than 10% of cells overlap. Our measurements indicate that this cell density is optimal to obtain an adequate number of comets for statistical analysis.

Our measurements using etoposide and electrochemical cell exposure are meant to provide examples of comet assay analysis typical of a treated cell population which can exhibit heterogeneous comet results. When used to analyze a set of cells that have undergone increasing levels of etoposide exposure our automated microscopy system provided an efficient tool for analysis of DNA fragmentation damage in the resulting cell populations. The graphical representation from the commercial imaging software provided a more thorough evaluation of individual comets. A plot of the statistical means of comet populations showed the expected increase in % DNA fragmentation on the level of the etoposide treatment (Fig. 2). When exposed to electrochemical oxidation, the automated analysis resulted in a distribution of cells with a wide range of DNA fragmentation, which is essential in order to evaluate the effects of a treatment that may result in significant cellular damage.

In summary, we conclude that an automated system of data collection and analysis, together with well characterized microscope and camera settings, is vital in order to properly quantify the DNA fragmentation from imaging analysis using the comet assay. Using an automated system of image analysis we examined the effect of varying camera exposure and focus and found limited ranges of camera settings that reduced a typical 40% variation in measured DNA fragmentation up to three-fold. Under these conditions, even a 50-fold variation in cell density yielded less than a 10% variation in the measured level of fragmentation. Although such settings must be determined individually for each specific microscope system, once they are established, proper measurements and differences inherent in the comet assay protocol (cell processing, electrophoresis, staining, etc.) can be properly accounted for.

## Additional Information

**How to cite this article**: Braafladt, S. *et al*. The Comet Assay: Automated Imaging Methods for Improved Analysis and Reproducibility. *Sci. Rep*. **6**, 32162; doi: 10.1038/srep32162 (2016).

## Supplementary Material

Supplementary Information

## Figures and Tables

**Table 1:** Comet Analysis of Cells after Electrochemical Treatment.

Sample Analyzed^a^	Mean (% DNA in Tail)	Stnd. Dev. (% DNA in Tail)	^#^Cells Analysed n
Control	7.96	7.01	373
Treated 1	31.6	29.3	166
Treated 2	34.7	38.0	121

^a^Treated 1 and Treated 2 were consecutive comet analyses performed on the same treated sample (6 h at 1V) using separately pipetted well plates.

**Table 2:** Comparison of comet % DNA determined at decreasing cell concentrations.

Parameter	Undiluted^a^	1:5 dilution	1:50 dilution
Mean (% DNA in Tail)	49.9	48.8	47.2
Stnd. Deviation^b^ (% DNA in Tail)	30.7	29.9	28.7
n^c^	72	125	36

^a^1 × 10^5^ to 2 × 10^5^ cells per milliliter (final concentration in agarose) were used in the comet procedure (see methods).

^b^Standard deviation of the mean % DNA in the tail of individual comets.

^c^Number of comets analyzed. Only non-overlapping comets were included in software analysis.
