# Supplementary material for: The Comet Assay: Automated Imaging Methods for Improved Analysis and Reproducibility
Source: Sci Rep. 2016 Sep 1;6:32162. doi: 10.1038/srep32162 (PMC5007470; doi:10.1038/srep32162)

Supplementary Information

The Comet Assay: Automated Methods for Quantitative Image Analysis and Reproducibility

Signe Braafladt, Vytas Reipa and Donald H. Atha

Biosystems and Biomaterials Division,

National Institute of Standards and Technology,

Gaithersburg, MD 20899

Supplementary Fig. 1: Imaging Software: Comparison of Image J and Commercial Software

A. Typical image outlining a single control used in the analysis of mean intensity.

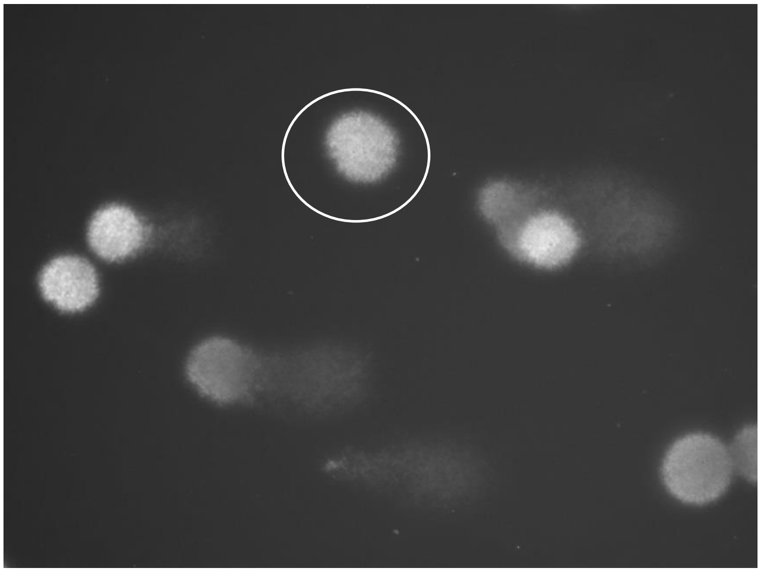

Supplementary Table1: Comparison of effective integrated intensity and the coefficient of variation (n=5) for analysis of a single control cell comet image.

Analysis Software	Mean Intensity [a] **X10^6**	Stnd. Dev. Inten.[b] **X 10^4**	% CV
Image J	1.88	2.35	1.25
Commercial	2.34	3.75	1.58

a. Images were captured using 5 s camera exposure.
b. Standard deviation of the mean of 5 measurements (n=5).

B. Typical image outlining a medium size tail comet used in the analysis of mean intensity and % DNA in tail.

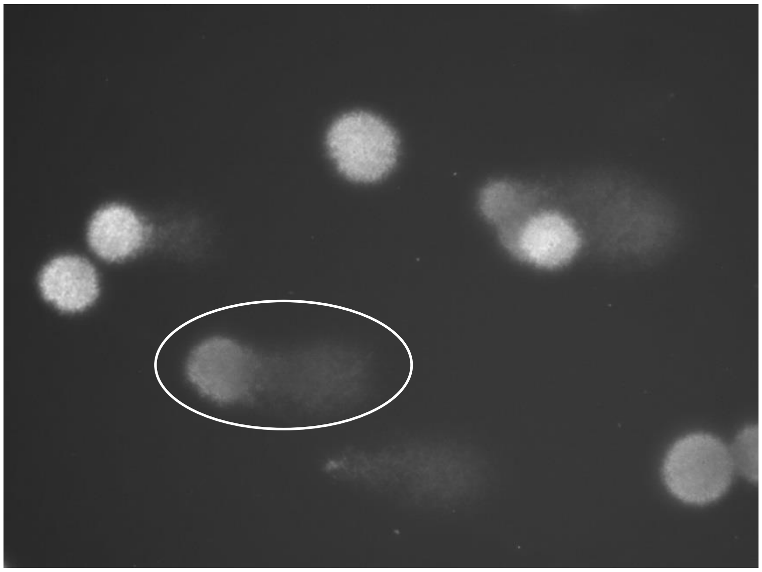

Supplementary Table 2: Comparison of % DNA in tail and coefficient of variation (n=5) for analysis of a single medium size tail comet image.

Analysis Software	Mean Intensity [a] x 10^5	Mean (% DNA in Tail)	Stnd.Dev.[b] (%DNA in Tail)	%CV (%DNA in Tail)
Image J	9.85	40.1	1.26	3.13
Commercial	13.5	31.7	1.76	5.56

a. Images were captured using 5 s camera exposure.
b. Standard deviation of the mean of 5 measurements (n=5).

Supplementary Fig. 2: Effect of camera exposure time on the integated intensity of a Single Comet Control Cell using Image J Software.

Plot of effective integrated intensity of a single control comet. The straight line is a least squares fit of the data with a correlation (R^2) of 0.9484.

The intensity threshold was adjusted so that both the medium and large tail comets were within the high and low background settings and the control cell (no strand breakage) only showed an integrated intensity measurement within the circular region of the nucleus. The integrated intensity of the single control comet was measured five times at each exposure and the average plotted as a function of camera exposure time.

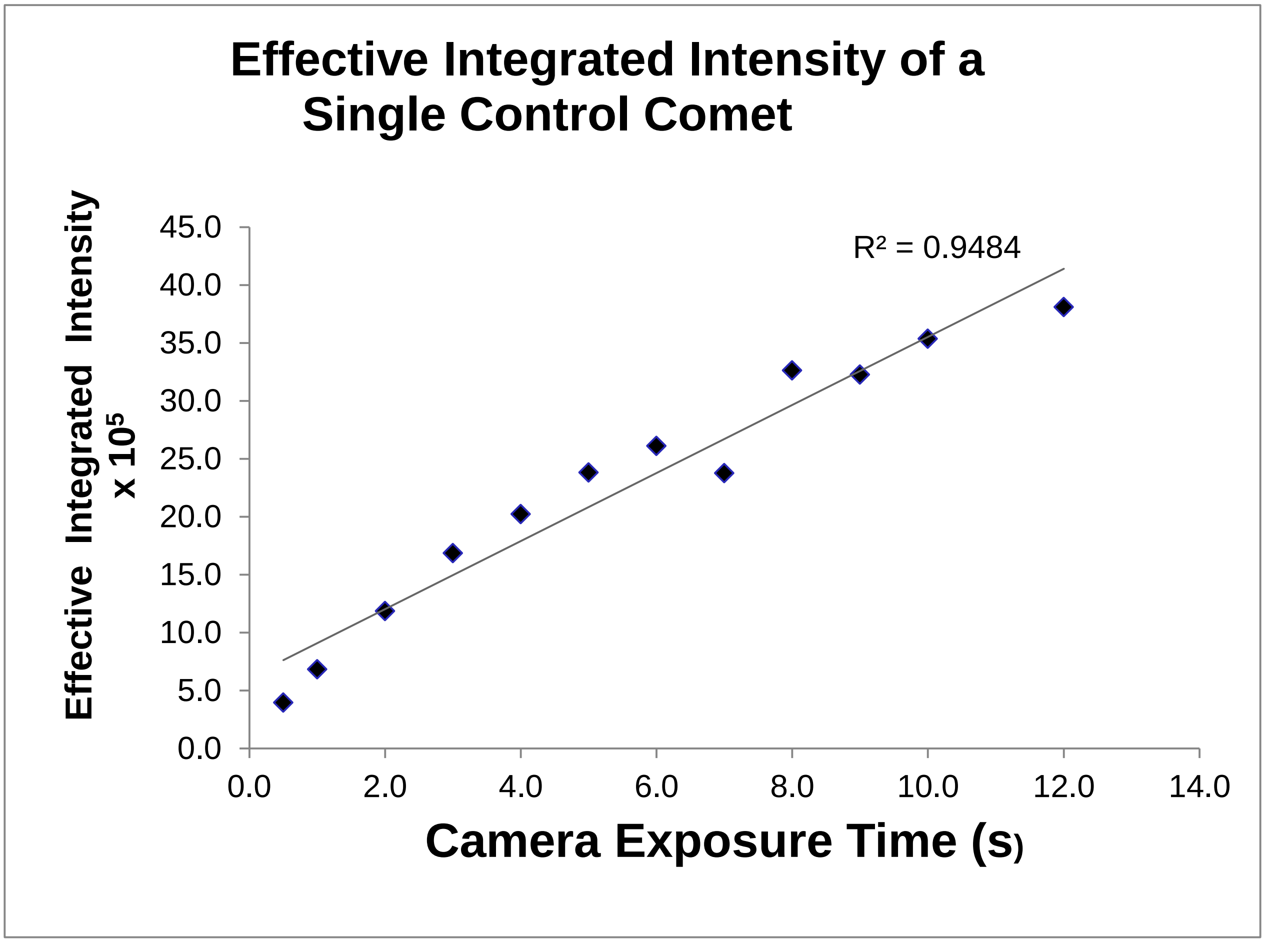

Supplementary Fig. 3: Effect of sample bleaching of fluorescence intensity – Light source blocked/unblocked between measurements.

Plot of measured integrated intensity as a function of time of exposure. The rate of bleaching is shown to be about one half when the light source is blocked between measurements. The effect of continuous camera exposure was examined to determine if significant bleaching of the Sybr Green dye would occurs during usual measurements (1 s to 12 s)

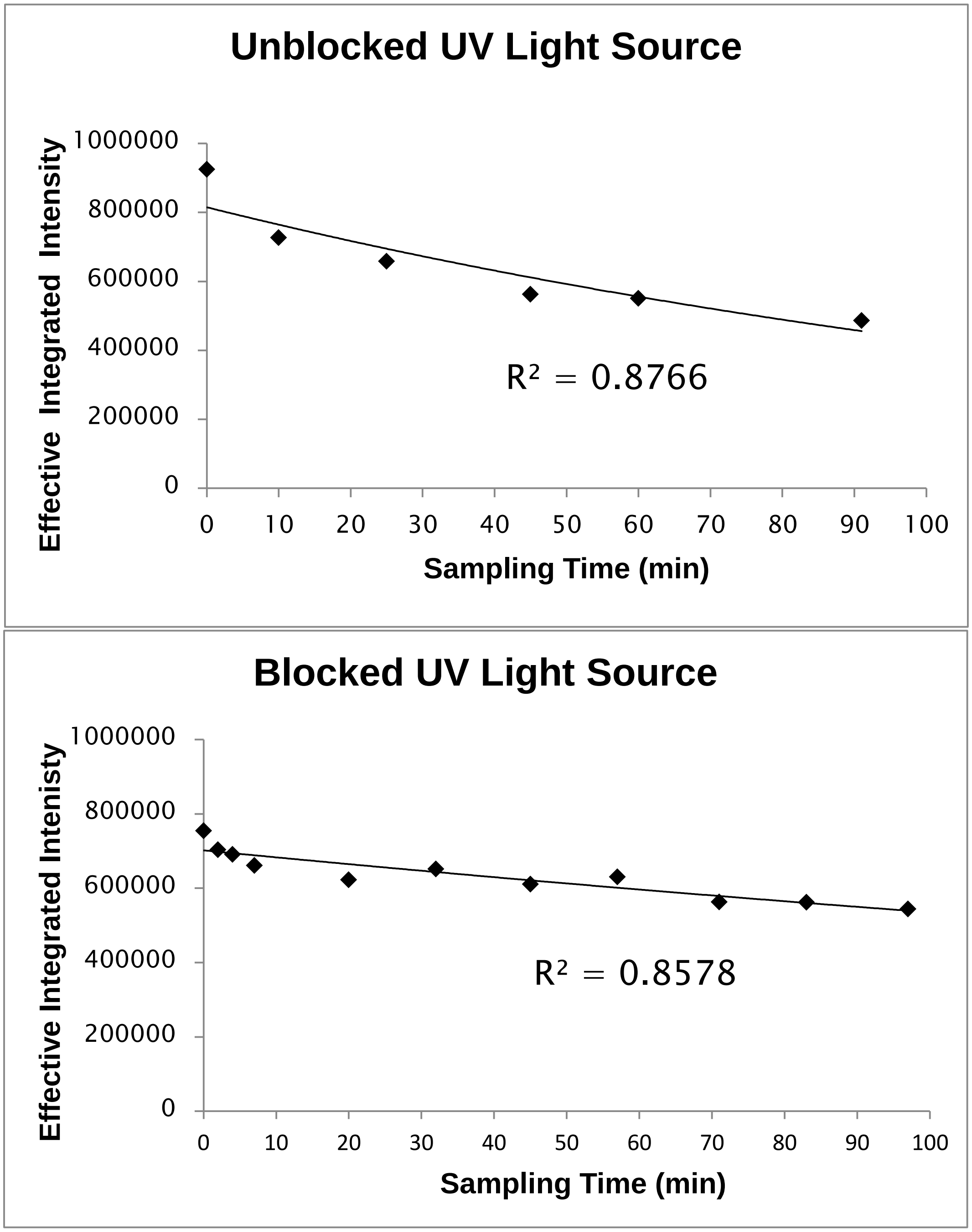

Supplementary Fig. 4: Slide Well-to-Well Reproducibility

Well-to-Well Reproducibility of Average % DNA in Tail.

Bar graph of reproducibility of average % DNA in tail as a function of treatment level. Error bars indicate standard deviation of the averages (n=5) for each level.

A twenty well slide was used for well-to-well comparison   Slide dimensions were 5 cm x 7.5 cm.

4 rows: CCO, CC1, CC2 and CC3 were used for increasing treatment level. 5 columns (n=5) were used for well-to-well comparison.

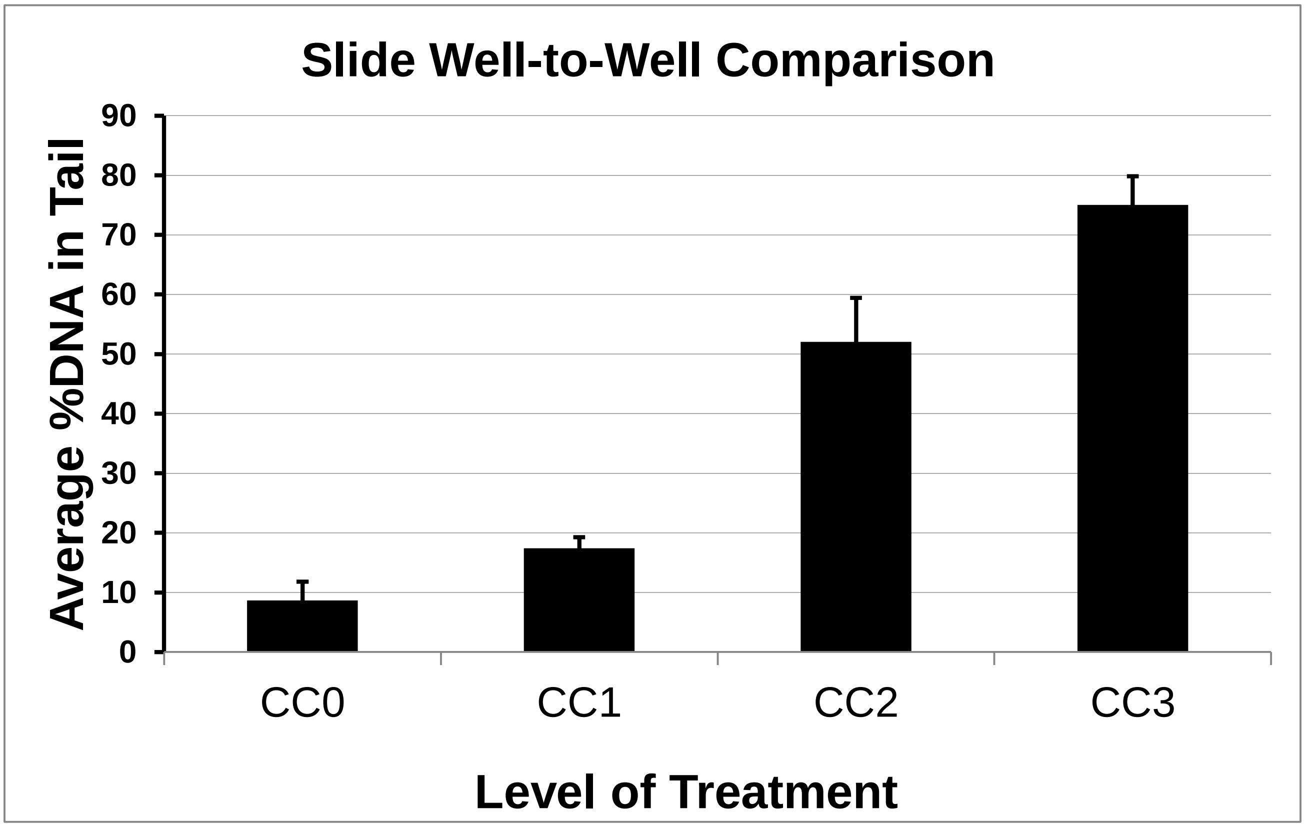

Supplement: Supplementary Information [file srep32162-s1.pdf]
